# WMDS.net: a network control framework for identifying key players in transcriptome programs

**DOI:** 10.1093/bioinformatics/btad071

**Published:** 2023-02-02

**Authors:** Xiang Cheng, Md Amanullah, Weigang Liu, Yi Liu, Xiaoqing Pan, Honghe Zhang, Haiming Xu, Pengyuan Liu, Yan Lu

**Affiliations:** Department of Gynecologic Oncology, Women’s Hospital and Institute of Translational Medicine, Zhejiang University School of Medicine, Hangzhou 310006, China; Institute of Bioinformatics, Zhejiang University, Hangzhou 310058, China; Institute of Bioinformatics, Zhejiang University, Hangzhou 310058, China; Department of Respiratory Medicine, Key Laboratory of Precision Medicine in Diagnosis and Monitoring Research of Zhejiang Province, Sir Run Run Shaw Hospital and Institute of Translational Medicine, Zhejiang University School of Medicine, Hangzhou 310016, China; Department of Respiratory Medicine, Key Laboratory of Precision Medicine in Diagnosis and Monitoring Research of Zhejiang Province, Sir Run Run Shaw Hospital and Institute of Translational Medicine, Zhejiang University School of Medicine, Hangzhou 310016, China; Institute of Bioinformatics, Zhejiang University, Hangzhou 310058, China; Department of Respiratory Medicine, Key Laboratory of Precision Medicine in Diagnosis and Monitoring Research of Zhejiang Province, Sir Run Run Shaw Hospital and Institute of Translational Medicine, Zhejiang University School of Medicine, Hangzhou 310016, China; Department of Mathematics, Shanghai Normal University, Xuhui 200234, China; Department of Pathology, Research Unit of Intelligence Classification of Tumor Pathology and Precision Therapy, Chinese Academy of Medical Sciences, Zhejiang University School of Medicine, Hangzhou 310058, China; Institute of Bioinformatics, Zhejiang University, Hangzhou 310058, China; Department of Gynecologic Oncology, Women’s Hospital and Institute of Translational Medicine, Zhejiang University School of Medicine, Hangzhou 310006, China; Department of Physiology, Center of Systems Molecular Medicine, Medical College of Wisconsin, Milwaukee, WI 53226, USA; Cancer Center, Zhejiang University, Hangzhou 310029, China; Department of Gynecologic Oncology, Women’s Hospital and Institute of Translational Medicine, Zhejiang University School of Medicine, Hangzhou 310006, China; Institute of Bioinformatics, Zhejiang University, Hangzhou 310058, China; Cancer Center, Zhejiang University, Hangzhou 310029, China

## Abstract

**Motivation:**

Mammalian cells can be transcriptionally reprogramed to other cellular phenotypes. Controllability of such complex transitions in transcriptional networks underlying cellular phenotypes is an inherent biological characteristic. This network controllability can be interpreted by operating a few key regulators to guide the transcriptional program from one state to another. Finding the key regulators in the transcriptional program can provide key insights into the network state transition underlying cellular phenotypes.

**Results:**

To address this challenge, here, we proposed to identify the key regulators in the transcriptional co-expression network as a minimum dominating set (MDS) of driver nodes that can fully control the network state transition. Based on the theory of structural controllability, we developed a weighted MDS network model (WMDS.net) to find the driver nodes of differential gene co-expression networks. The weight of WMDS.net integrates the degree of nodes in the network and the significance of gene co-expression difference between two physiological states into the measurement of node controllability of the transcriptional network. To confirm its validity, we applied WMDS.net to the discovery of cancer driver genes in RNA-seq datasets from The Cancer Genome Atlas. WMDS.net is powerful among various cancer datasets and outperformed the other top-tier tools with a better balance between precision and recall.

**Availability and implementation:**

https://github.com/chaofen123/WMDS.net.

**Supplementary information:**

Supplementary data are available at *Bioinformatics* online.

## 1 Introduction

In biological processes, genes and proteins rarely act alone, they rather act in concert and in parallel to form a complex network-like interactome (Barabasi *et al.*, 2011). The irregular perturbation of this interactome can lead to the pathological state, such as cancer (Grechkin *et al.*, 2016). Transcriptional co-expression network is one of key interactomes in eukaryote, where the nodes represent genes and edges between genes correspond to significant interactive relations (Barabasi and Albert, 1999). This type of network can be constructed from genome-wide expression profiling that is carried out by microarray, RNA-seq and other high-throughput technologies (Basso *et al.*, 2005; Delgado and Gomez-Vela, 2019; Kuzmanovski *et al.*, 2018). It represents the concerted gene regulation programs through statistical inference of co-expression patterns (de Anda-Jáuregui *et al.*, 2019; Langfelder and Horvath, 2008). The study of transcriptional co-expression network can help to understand the key cellular behavior from a systems perspective (Eisenberg *et al.*, 2000).

During different developmental stages, the transition of different cell states, or the progression from a normal state to a disease state, living cells often undergo drastic rewiring of the transcription co-expression networks (Flintoft, 2004). Notably, the rewiring of transcriptional networks in living cells not only involves in the destruction of existing connectivity between genes, but also the reconstruction of new connectivity between genes. Several methods have been developed to identify differential co-expression networks under different conditions, such as developmental stages and disease status (Barabasi *et al.*, 2011; Ideker and Krogan, 2012). However, most of these previous methods focused on finding co-expression patterns varying between different conditions (de la Fuente, 2010; Gustafsson *et al.*, 2014; Lu *et al.*, 2007). There are very few methods to detect which key players in the transcriptional network drive cells from one state to another.

Mammalian cells can be transcriptionally reprogramed to other cellular phenotypes (Huang *et al.*, 2011; Ieda *et al.*, 2010; Szabo *et al.*, 2010; Takahashi and Yamanaka, 2006; Vierbuchen *et al.*, 2010). Controllability of such complex transitions in transcriptional networks underlying cellular phenotypes seems to be an inherent biological characteristic and is used to develop new disease models, tissue engineering and stem cell therapies (Muller and Schuppert, 2011). According to the structural controllability theory, the ability to steer a complex network from any initial state to any desired state can be measured by the minimum number of driver nodes required to input external signals, thereby completely controlling the whole network (Hu *et al.*, 2019; Liu *et al.*, 2011). The maximum matching approach (Liu *et al.*, 2011) has been used to identify driver nodes in directed networks, while the minimum dominating set (MDS) (Nacher and Akutsu, 2012) was also recently proposed to identify driver nodes in undirected networks. When each edge in a network is bi-directional, and each node in the MDS can individually control all of its outgoing links, this bi-directional network is structurally controllable by selecting the nodes in the MDS as driver nodes. In this case, this MDS can be regarded as a maximal matching. To investigate transcriptional regulation, we proposed to identify the key players in the transcriptional network as a MDS of driver nodes that can fully control the network state transition. However, different MDSs will be predicted in the same network because the MDS model is not unique. Therefore, we employed a weighted minimum dominating set (WMDS) network model (WMDS.net) to determine the driver nodes of network. The weight of WMDS.net integrates the degree of nodes in the network and the significance of gene co-expression difference between two states into the measurement of node controllability of the transcriptional network.

To illustrate the utility of WMDS.net, we applied it to the discovery of cancer driver genes in RNA-seq data from The Cancer Genome Atlas (TCGA). Driver genes modify transcriptional programs in the process of tumorigenesis and cancer progression, driving and sustaining a biological system from healthy state to disease state, whereas most of altered genes detected in cancer genomics studies are passengers that are irrelevant to tumor development and progression (Greenman *et al.*, 2007; Han *et al.*, 2019; Hua *et al.*, 2013). In this application, we aimed to identify critical nodes of the transcriptional co-expression network constructed from RNA-seq data of tumor and adjacent normal tissues, which guide cells from normal to hyper-proliferative state. We further compared our method with other 11 top-tier driver gene detection methods on 14 TCGA datasets (Bashashati *et al.*, 2012; Gonzalez-Perez and Lopez-Bigas, 2012; Guo *et al.*, 2018, 2019; Han *et al.*, 2019; Hou and Ma, 2014; Hua *et al.*, 2013; Jia *et al.*, 2014; Lawrence *et al.*, 2013; Reimand and Bader, 2013; Ryslik *et al.*, 2013). Our comprehensive evaluation demonstrated that WMDS.net was powerful among various datasets and outperformed the other top-tier tools. Importantly, WMDS.net can effectively identify driver genes of low-frequency mutations in tumor samples and reveals the unexpectedly high tumor heterogeneity of cancer patients in the same type of cancer and thus have important implications in personalized treatment for cancer patients.

## 2 Materials and methods

### 2.1 Gene-interaction reference network

We used the gene-interaction reference network constructed in Dawnrank (Hou and Ma, 2014), which integrated several sources, including the network used in MEMo (Ciriello *et al.*, 2012), the curated information from Reactome (Croft *et al.*, 2011), the NCI-Nature Curated PID (Schaefer *et al.*, 2009) and KEGG (Kanehisa *et al.*, 2012). The MEMo network contained inferred gene interactions, such as protein interactions, gene co-expression and protein domain interactions. There are 11 648 genes and 211 794 edges in the resulting aggregated reference network. Then, we selected the largest connected subgraph with the most nodes among all the connected subgraph of the graph as the central part of the network. Genes that have no edge connection with the central part of the network were deleted, only the central part of the network as a whole network was retained in the collected gene-interaction reference network. Because these scattered nodes without interaction with the central part are separated from most of the nodes in the network, their control impacts on the whole network are negligible.

### 2.2 Multiple-sample differential co-expression network

**Fig. 1.**
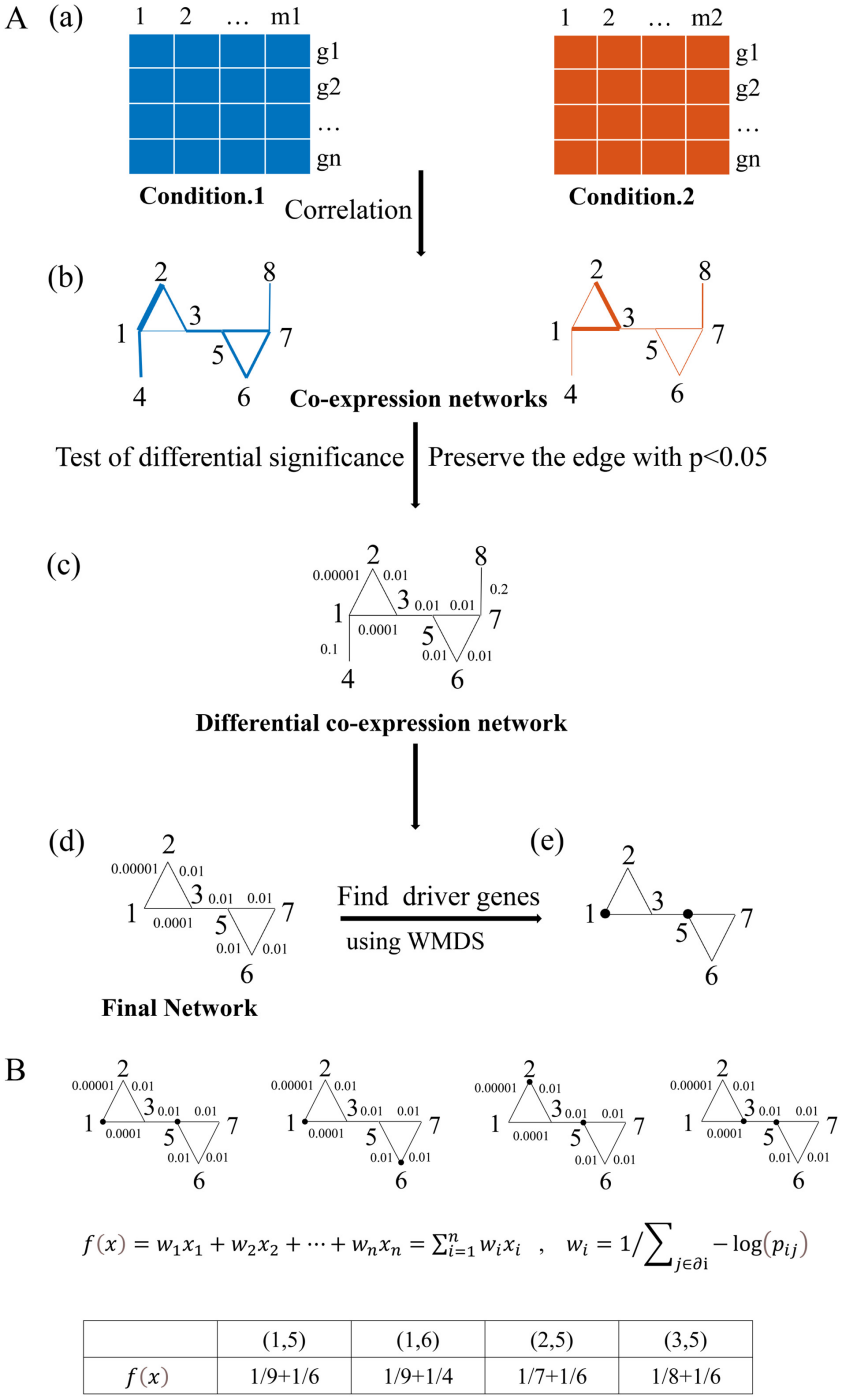
Overview of WMDS.net. (**A**) Flowchart of WMDS.net. (a) Expression matrices of samples under two conditions. (b) Co-expression network. Co-expression networks for two conditions were constructed based on the reference gene-interaction network, respectively. Each node of the co-expression network represents a gene, and the thickness of each edge represents the magnitude of Pearson correlation coefficient of two genes. (c) Differential co-expression network. The number next to the edge of the differential co-expression network represents the *P*-value of the significance test of the differences between the edges in two states. (d) Final network. Only the edges with *P*-value <0.05 within the reference gene-interaction network were preserved. (e) Identification of driver nodes in the final differential expression network. WMDS was applied to find the driver genes of the final network, which are represented by black spots. (**B**) WMDS model. The DS is defined such that each node in the graph is either a point in DS or an adjacent to an element of DS. A MDS is the smallest DS for a given network. A set of black points in the upper panel represents an MDS in a given network. As the MDS is not unique in the whole network, we applied the WMDS model instead in which the weight integrates the degree of nodes in the network and the significance of gene co-expression difference between two states. For an intuitive illustration of WMDS, γ was set to 1. Based on the objection function, f(x), in the lower panel, (1, 5) is an optimal MDS for the studied network

To quantify the edge between genes in co-expression networks, the Pearson correlation coefficients of mRNA expression profiles were used to calculate the expression correlation between genes connected in the reference network (Fig. 1A). The Pearson correlation between expression levels of two genes ($x_{i}$, $x_{j}$) can be quantified:

(1) $$r_{ij}=\mathrm{cov}\left(x_{i},x_{j}\right)/\sqrt{\mathrm{var}\left(x_{i}\right)v\mathrm{ar}\left(x_{j}\right)},$$

where the cov represents the covariance of expression levels between two genes ($x_{i}$, $x_{j}$) connected in the reference network and the var represents the variance of expression levels of genes $x_{i}$ and $x_{j}$. mRNA expression for Pearson correlation analysis was quantified using Fragments Per Kilobase of transcript per Million mapped reads.

The correlation of expression levels between a pair of genes ($x_{i}$, $x_{j}$) in one condition was recorded as $r_{ij}^{1}$ and in another condition was recorded as $r_{ij}^{2}$. The significance of differential co-expression (i.e. differential edge) between two conditions was examined using *Z*-test. Briefly, the Fisher *Z*-transformation was performed to convert correlation coefficient into an approximately normally distributed measure (Bhuva *et al.*, 2019):

(2) $$z_{ij}^{k}=\frac{1}{2}\ln\left|\frac{1+r_{ij}^{k}}{1-r_{ij}^{k}}\right|,k=1,2,$$

where $z_{ij}$ was obtained by the Fisher *Z*-transformation of $r_{ij}$; $z_{ij}$ is asymptotically the normal distribution with standard deviation of $\sqrt{\frac{1}{n-3}}$ (Fisher, 1915; Torres, 2020), where n is the sample size. That is,

(3) $$z_{ij}^{1}\;∼\;N\left(\rho_{1},\frac{1}{n_{1}-3}\right)$$

and

(4) $$z_{ij}^{2}\;∼\;N\left(\rho_{2},\frac{1}{n_{2}-3}\right),$$

where $n_{1}$ and $n_{2}$ are the sample sizes of two conditions, respectively. Under the null hypothesis that the correlation coefficients of the two population samples are equal (i.e. $\rho_{1}=\rho_{2}$), we could derive the following statistic,

(5) $$\Delta z_{ij}=\frac{\left|z_{ij}^{1}-z_{\dot{l}j}^{2}\right|}{\sqrt{\frac{1}{n_{1}-3}+\frac{1}{n_{2}-3}}}\;∼\;N\left(0,1\right).$$

The above statistic $\Delta z_{ij}$ is asymptotically the normal distribution with a mean of zero and a standard deviation of 1.

To assess the influence of sample sizes on the construction of differential co-expression networks, we simulated the distribution of ΔZ under the null hypothesis by randomly generating two pairs of sequences with sample size of 5, 8, 10, 15 and 20, 100 for 2 000 000 times. Kolmogorov–Smirnov test was used to examine whether the simulated distribution is identical to standard normal distribution. As shown in Supplementary Figure S1, the distribution of ΔZ is a good approximation of standard normal distribution when the sample size is >15. In other words, *P*-values estimated for differential edges between two conditions are valid for sample size as small as 15.

### 2.3 Personalized differential co-expression network

Considering the individual heterogeneity of a certain type of cancer, we also constructed personalized differential co-expression networks using the sample specific network (SSN) (Liu *et al.*, 2016). For a group of normal samples (n samples), the correlation $r_{ij}^{n}$ of a pair of genes was calculated according to the expression data of these samples to construct the co-expression reference network. In general, the normal sample reference network contains the common properties of these normal samples. Next, an individual tumor sample from a cancer patient was added to the reference samples, and the correlation $r_{ij}^{n+1}$ of a pair of genes was re-calculated to construct a co-expression perturbed network due to this additional tumor sample. The difference between the corresponding edges of co-expression reference network and the co-expression perturbed network, i.e. $\Delta r_{ij}=r_{ij}^{n+1}-r_{ij}^{n}$, was calculated. Then, $\Delta r_{ij}$ was transformed into *Z*-score, and *P*-value for $\Delta r_{ij}$ was obtained from *Z*-test. If *P*-value <0.05, the edge $r_{ij}$ is retained for that patient (Supplementary Fig. S2). Finally, we predicted personal driver genes for each cancer patient on their personalized differential co-expression network using WMDS.

### 2.4 WMDS model

The definition of MDS is as follow. Given a gene network, which can be represented by a graph G=(V,E), where V is the set of gene nodes and E is the set of edges between genes, if each gene v∈V is an element of the DS set or is adjacent to an element in the DS set, DS is a dominating set (Nacher and Akutsu, 2012). The MDS is the minimum DS of the gene network (Fig. 1B). Since the dominating problem is a classical non-deterministic polynomial-hard problem in computational complexity theory (Haynes *et al.*, 1998), there is no polynomial time algorithm to find the MDS of arbitrary graphs. Therefore, we simplified the dominating problem to a binary integer programming problem. Binary integer programming is a mathematic algorithm for finding a binary vector x, which minimizes the linear function f(x) subject to linear constraints. Each node i is assigned to a binary integer variable $x_{i}$ taking 0 or 1. If node i belongs to the MDS, then $x_{i}$ takes 1, otherwise takes 0. To calculate MDS, we considered the minimization of the following linear functions:

(6) $$f\left(x\right)=x_{1}+x_{2}+\cdots +x_{n}=\sum_{i=1}^{n}x_{i}$$

subject to the constraints:

$$x_{i}+\sum_{j\in \partial i}x_{j}\geq 1$$

where ∂i is the adjacent node of i and n is the total number of nodes in the network.

As shown in Figure 1B, there may be more than one optimal solution for the binary integer programming problem in a given network. It is challenging to determine which one is the real set of critical nodes that controls the whole biological network. To solve this problem, we applied a method similar to that of Bakhteh *et al.* (2020) to compute a heuristic approximation of the weighted minimum dominating set (WMDS) (Garey and Johnson, 1990), where biologically meaningful information was incorporated into the weights. This weighted approach allows us to select an MDS configuration that best represents a set of key nodes that likely govern the entire differential co-expression network. Firstly, the driver nodes often show high connectivity in the gene network (D'Antonio and Ciccarelli, 2013; Khurana *et al.*, 2013). For gene-interaction networks, it is known that cancer driver genes tend to have high degree (Schaefer *et al.*, 2015). Thus, we set the weight of the nodes as degree of the nodes of differential co-expression network. Secondly, the significant difference in the co-expression correlation of genes between two conditions indicates that these genes may play an important role in steering the network from one state to another state. Living cells often undergo dramatic rewiring of transcriptional co-expression networks during different developmental stages, transitions between different cellular states, or progression from normal to disease states (Flintoft, 2004). Molecular disease phenotypes typically constitute abnormalities in the co-regulation of genes (Golub *et al.*, 1999). Studies also showed that disruption of co-expression among genes is prevalent during tumorigenesis (Deng *et al.*, 2015; Lin *et al.*, 2015). Therefore, we further considered the edge weight that represents the confidences of the interaction between genes in the differential co-expression network. We hypothesized that genes with high degree of connectivity and significant correlation difference are more likely to become key players in the network state transition. Therefore, we defined the weight $w_{i}={（\sum_{j\in \partial i}-\log\left(p_{ij}\right)）}^{-\gamma }$ of each gene *i*, where ∂i is the set of adjacent genes of gene i; $p_{ij}$ is the *P*-value of significant difference in the co-expression correlation of gene i and adjacent gene j between the two states; γ (> 0) is a hyperparameter controlling the weights (Zhang *et al.*, 2015) to ensure that the number of nodes in weighed dominating set (WDS) is the same as in all MDS configurations, thereby minimizing the number of nodes in WDS. In other words, we ensure that the WDS is a configuration choice among all MDS configurations, i.e. it becomes WMDS. If γ =  0, then $w_{i}=1$, i.e. there is no weight for each node. We set γ=0.01 in our WMDS model.

Then, the binary integer programming problem is transformed into the minimization of the following linear functions:

(7) $$f\left(x\right)={w_{1}x}_{1}+{w_{2}x}_{2}+\cdots +w_{n}x_{n}=\sum_{i=1}^{n}w_{i}x_{i}$$

subject to the constraints:

$$x_{i}+\sum_{j\in \partial i}x_{j}\geq 1$$

where $w_{i}$ is the weight of gene i and $x_{i}$ takes 1 or 0. Among all the MDS configurations, we tended to select the MDS whose members have the highest degree and most significant difference of co-expression correlation in a given network. For example, in Figure 1B, several optional gene sets, such as $\left(1,5\right),\;\left(1,6\right),\;\left(2,5\right)\;\mathrm{and}\;(3,5)$, can be selected as the driver node set of the network by applying MDS model. If the WMDS model is applied, the weight of (1,5) in these optional choices is the minimum. Taken together, the WMDS model tends to predict genes with high degree of connectivity and significant difference of co-expression correlation in a given network as driver nodes. The source codes to implement WMDS.net are available at https://github.com/chaofen123/WMDS.net.

### 2.5 *F*-measure evaluation criteria

*F*-measure was used to evaluate the efficiency of WMDS.net and other driver prediction tools, which is the weighted harmonic mean of precision and recall with non-negative weight β. *F*-measure was calculated as follows:

$$\begin{array}{c} \mathrm{Precision}\;=\frac{\mathrm{true}\;\mathrm{positive}}{\mathrm{true}\;\mathrm{positive}+\mathrm{false}\;\mathrm{positive}}\\ \begin{array}{c} \mathrm{Recall}\;=\frac{\mathrm{true}\;\mathrm{positive}}{\mathrm{true}\;\mathrm{positive}+\mathrm{false}\;\mathrm{negative}}\\ F_{\beta }=\frac{\left(1+\beta^{2}\right)\mathrm{Precision}\;\times \;\mathrm{Recall}}{\beta^{2}\mathrm{Precision}\;+\;\mathrm{Recall}} \end{array} \end{array}$$

where $\beta^{2}$ is generally taken as the value of 0.3, which is based on the experience of many target detection works (Achanta *et al.*, 2009; Cheng *et al.*, 2015; Perazzi *et al.*, 2012). In other words, the weight of precision is promoted since the precision is considered more important than the recall in real experiments. In the evaluation of cancer driver gene prediction, we found that $\beta^{2}$ taken with the value of 0.2 is objective and fair, according to the total number of genes on the genome and the number of driver genes in the standard gene list. If we take $\beta^{2}=1$ (denoted as *F*1-measure), which was used by some previous tools (Guo *et al.*, 2019), i.e. the importance of precision and recall is the same. However, even if nothing is done, the *F*1-measure is not low with all genes predicted as driver genes in which the precision is very low, but the recall will be equal to 1. For example, there are 616 genes in the cancer gene census (CGC) gene list, and the prediction of all 25 000 genes on the genome as driver genes is more efficient than some existing tools (e.g. DriverML and DawnRank) with excellent precision according to the *F*1-measure. Therefore, we analyzed the two worst cases: (i) all 25 000 genes are predicted as driver genes when the recall is 1, and (ii) only 2∼3 genes are predicted as driver genes when the precision is 1. In order that the evaluation criteria will not be biased toward tools with more or less predictions, we chose an appropriate $\beta^{2}$ (i.e. a value of 0.2) so that the *F*-measures of the two worst cases are equal. In addition, the *F*1-measure (i.e. $\beta^{2}=1$) was provided in the Supplementary Material.

### 2.6 Data collection

The mRNA expression profiles of 14 types of common cancer from TCGA were used for identifying driver genes, each of which contained more than 15 normal samples (Supplementary Table S1). These cancer types included bladder urothelial carcinoma (BLCA), breast invasive carcinoma (BRCA), colon adenocarcinoma (COAD), head and neck squamous cell carcinoma (HNSC), kidney chromophobe (KICH), kidney renal clear cell carcinoma (KIRC), kidney renal papillary cell carcinoma (KIRP), liver hepatocellular carcinoma (LIHC), lung adenocarcinoma (LUAD), lung squamous cell carcinoma (LUSC), prostate adenocarcinoma (PRAD), stomach adenocarcinoma (STAD), papillary thyroid carcinoma (THCA)) and uterine corpus endometrial carcinoma (UCEC). Gene expression and single nucleotide variation (SNV) data were downloaded from the Genomic Data Commons (https://portal.gdc.cancer.gov). The gene list of CGC was downloaded from the COSMIC website (https://cancer.sanger.ac.uk/cosmic). The gene list of network of cancer genes (NCG) was downloaded from the web resource (http://ncg.kcl.ac.uk/download.php). The gene lists of Pan-cancer drivers, Mut-drivers and Hiconf drivers were extracted from previous studies (Kumar *et al.*, 2015; Tamborero *et al.*, 2013a; Vogelstein *et al.*, 2013) and merged into one list (denoted as PMH).

### 2.7 Other tools for predicting cancer driver genes

In the application of detecting cancer driver genes, we compared our methods with other 11 top-tier tools in the TCGA datasets. These tools detect driver genes either by somatic mutation frequency, or gene expression, or gene or protein interaction networks. This allows us to conduct an unbiased and comprehensive evaluation of our newly proposed WMDS.net. They included DrGap (Hua *et al.*, 2013), OncoDriveFM (Gonzalez-Perez and Lopez-Bigas, 2012), MutSigCV (Lawrence *et al.*, 2013), DriverML (Han *et al.*, 2019), PNC (Guo *et al.*, 2019), DawnRank (Hou and Ma, 2014), SCS (Guo *et al.*, 2018), DriverNet (Bashashati *et al.*, 2012), ActiveDriver (Reimand and Bader, 2013), iPAC (Ryslik *et al.*, 2013) and MSEA (Jia *et al.*, 2014). Predictions of driver genes in the other tools were obtained from the DriverDBv2 database (Chung *et al.*, 2016).

## 3 Results

### 3.1 Overview of WMDS.net

WMDS.net is an innovative network control approach to identify key players in gene co-expression networks (https://github.com/chaofen123/WMDS.net). It mainly consists of two steps (Fig. 1A). First, we obtained a priori gene-interaction network that contains highly credible interactions between gene pairs from various data resources (Ciriello *et al.*, 2012; Croft *et al.*, 2011; Hou and Ma, 2014; Kanehisa *et al.*, 2012; Schaefer *et al.*, 2009). From this reference network, we constructed the co-expression networks of two different conditions (e.g. normal state and tumor state) using high-throughput expression profiles, and further integrated the two networks into the final differential co-expression network. Second, we employed the WMDS network model (Fig. 1B) to predict driver nodes that can steer the transcriptomic biological network from one state to another. The transcriptional co-expression network can be represented as an undirected graph, in which the graph nodes correspond to genes and edges between genes correspond to significant interactive relations. Then, we applied WMDS to find the driver nodes in the whole network. In the WMDS model, the weight integrates the degree of nodes in the network and the significance of gene co-expression difference between two conditions. In the context of cancer, we could also construct individualized differential co-expression networks using the SSN (Liu *et al.*, 2016) (Supplementary Fig. S2) to find the cancer patient-specific driver gene set.

### 3.2 Construction of differential co-expression networks in TCGA datasets

**Fig. 2.**
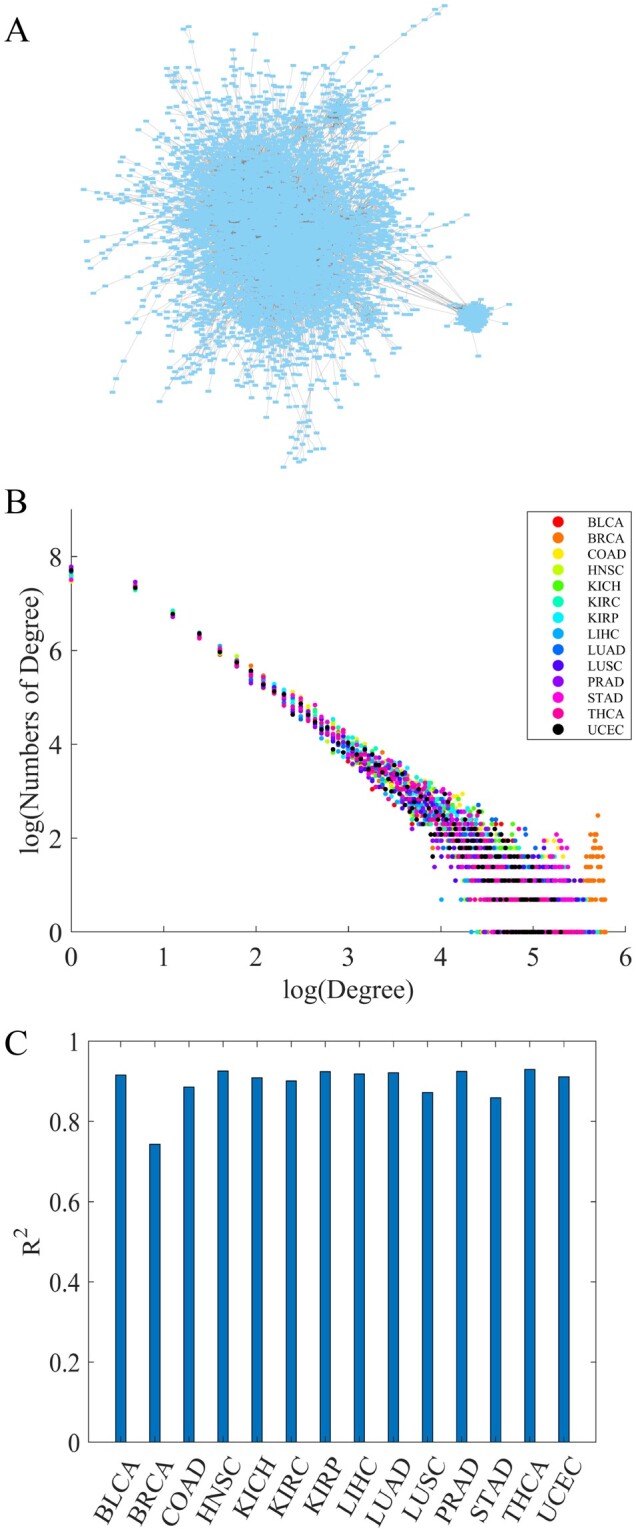
Differential gene co-expression networks. (**A**) Final transcriptome network constructed by WMDS.net. There were 8745 genes and 174 923 edges in the resulting aggregated network in BRCA. (**B**) Overall degree distribution of network nodes from 14 TCGA datasets. In each cancer type, log2(numbers of degree) versus log2 (degree) for the constructed differential co-expression networks was plotted. (**C**) *R*^2^ of the fitting linear regression model with log2(numbers of degree) as a response variable and log2 (degree) as an independent variable in each cancer type. *R*^2^ quantifies how well a network satisfies a scale-free topology

Driver nodes of transcriptional networks in tumor cells are potential cancer driver genes that play key roles in reprograming tumor cellular phenotypes from a normal state to hyper-proliferative state. To illustrate the utility of WMDS.net, we applied it to analyze RNA-seq data to predict cancer driver genes in TCGA. We thus constructed differential co-expression networks in 14 major types of cancer from TCGA (Supplementary Table S1) each of which had at least 15 normal samples (Fig. 2A). Most biologically relevant networks are scale free, which consist of many lowly connected genes and a small number of highly connected ‘hub’ genes (Basso *et al.*, 2005; van Noort *et al.*, 2004). This kind of biological network often have a power-law distribution (Barabasi and Albert, 1999), i.e. the frequency distribution of degree follows $p\left(k\right)∼k^{-\gamma }$. To visually inspect whether an approximate scale-free topology is satisfied, we plotted log2(numbers of degree) versus log2 (degree) for the constructed differential co-expression networks of all 14 cancer types in TCGA (Fig. 2B and Supplementary Fig. S3). The straight line is indicative of scale-free topology. The fitting index *R*-square ($R^{2}$) of linear regression model can be used to quantify how well a network satisfies a scale-free topology, and $R^{2}>0.80$ is a better approximation (Zhang and Horvath, 2005). As shown in Figure 2C, $R^{2}$ on most TCGA datasets are >0.9, indicating that our differential co-expression networks satisfy the approximate scale-free topology well. Therefore, these differential co-expression networks constructed from TCGA datasets are of biological significance and are suitable for finding driver nodes that control the whole transcriptome network switching between the normal and tumor states.

### 3.3 Performance evaluation of WMDS.net in predicting cancer driver genes

To evaluate the efficiency of our method in predicting cancer driver genes, we collected 616 and 711 cancer-associated genes from the CGC (https://cancer.sanger.ac.uk/cosmic) and NCG (http://ncg.kcl.ac.uk/download.php) databases, respectively. The true cancer driver genes are not known, however, well curated cancer gene databases, such as CGC and NCG, provide approximate benchmarks for true cancer drivers. The CGC database manually collected a set of genes whose mutations have a causal relationship with cancer. The NCG database collected a comprehensive catalogue of known and candidate cancer genes from various tumor genome sequencing projects. In view of the potential biased nature of CGC and NCG databases and the fact that many cancer genes have not yet been discovered, the researchers identified several additional sets of putative cancer genes from other data sources. Tamborero *et al.* identified mutational cancer driver genes across 12 cancer types (denoted as Pan-cancer drivers) from TCGA (Tamborero *et al.*, 2013a). Genes in the Pan-cancer list were predicted as cancer drivers by at least three of the four following tools: MuSiC (Dees *et al.*, 2012), OncodriveFM (Gonzalez-Perez and Lopez-Bigas, 2012), OncodriveCLUST (Tamborero *et al.*, 2013b) and ActiveDriver (Reimand and Bader, 2013). Using the ‘20/20’ rule, Vogelstein *et al.* reported a list of Mut-driver genes that were determined by their mutation patterns rather than mutation frequencies (Vogelstein *et al.*, 2013). In addition, Kumar *et al.* identified a set of high confident (Hiconf) cancer genes through comprehensive literature search (Kumar *et al.*, 2015). We integrated the last three sets of cancer-associated genes (i.e. Pan-cancer, Mut-driver and Hiconf) into one gene set (denoted as PMH), which includes 501 genes and is different mostly from the CGC gene list.

In the following, we compared the performance of our method with other 11 top-tier tools (Bashashati *et al.*, 2012; Gonzalez-Perez and Lopez-Bigas, 2012; Guo *et al.*, 2018, 2019; Han *et al.*, 2019; Hou and Ma, 2014; Hua *et al.*, 2013; Jia *et al.*, 2014; Lawrence *et al.*, 2013; Reimand and Bader, 2013; Ryslik *et al.*, 2013) in various TCGA datasets based on the above three benchmarks for cancer driver genes (i.e. CGC, NCG and PMH). These selected tools not only perform well, but also are widely used in the field. Furthermore, these tools are based on different principles to detect driver genes, such as through somatic mutation frequency, gene expression or gene or protein interaction network. This will help us to make an unbiased and comprehensive assessment of our newly proposed WMDS.net. F0.2-measure evaluation criteria were used to assess the performance of these driver gene prediction tools in 14 TCGA datasets. F0.2-measure is a comprehensive indicator that simultaneously evaluates accuracy and recall from a balanced perspective.

In each dataset, our method predicted two lists of cancer driver genes using the WMDS network control algorithm. One was based on the multi-sample differential co-expression network (denoted as WMDS.net). WMDS.net can capture the transition between the normal state and the tumor state of a specific type of cancer at the global level. We also wanted to evaluate the performance of our method at the individual patient level. Therefore, the other one was based on the single patient-specific personalized differential co-expression network (denoted as WMDS.netP). In the WMDS.netP method, each cancer patient yielded a list of personalized driver genes. For fair comparison with other driver prediction tools, if the frequencies of the patient’s personalized driver genes in the specific cancer type were ≥0.5, they were merged into a final list. The resulting final list represents driver genes of this cancer type that were predicted at the global level.

**Fig. 3.**
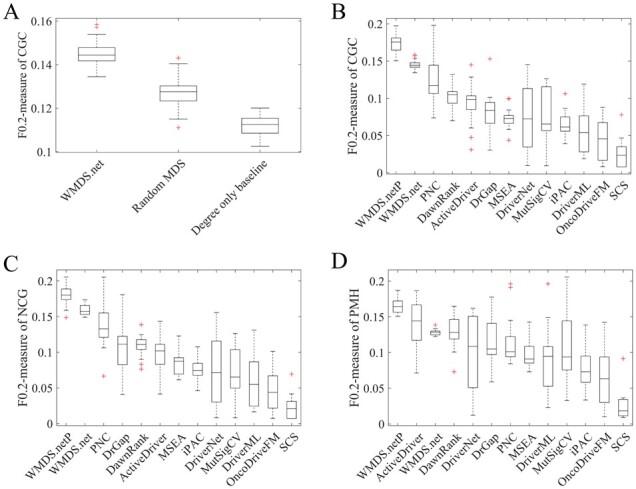
F0.2-measure of predicted driver genes in 14 TCGA datasets. (**A**) Comparisons of our method with an unweighted method and a naive ‘degree only’ baseline. (**B**) CGC benchmark. (**C**) NCG benchmark. (**D**) PMH benchmark. Tools were ordered by their median F0.2-measure of predicted drivers in each benchmark among 14 cancer types

We first compared WMDS.net with an unweighted MDS method, where one of MDS configurations was randomly selected to represent critical nodes of the differential co-expression network. As shown in Figure 3A, compared with the unweighted MDS, incorporating biologically meaningful weights significantly improved the performance of our method in predicting cancer driver genes. Next, we compared WMDS.net with a naive ‘degree only’ baseline method that ranks genes by their vertex score (Fig. 3A). For a fair comparison, we selected the same number of driver genes predicted by the ‘degree only’ baseline method as WMDS.net. This comparison showed that WMDS.net performed much better than the naive ‘degree only’ baseline largely due to the use of MDS in the differential co-expression network.

Then, we compared the performance of our method with 11 other tools in three benchmarks. In the CGC benchmark, WMDS.netP and WMDS.net achieved the two highest F0.2-measure values, ∼30% higher than PNC that ranked third (Fig. 3B and Supplementary Fig. S4). Notably, WMDS.net not only produced high F0.2-measure values, but also produced less variation in F0.2-measure values. In the NCC benchmark, WMDS.netP and WMDS.net also performed best among these driver gene prediction tools (Fig. 3C and Supplementary Fig. S5). In the PMH benchmark, WMDS.netP, ActiveDriver and WMDS.net ranked in the top three tools according to F0.2-measure (Fig. 3D and Supplementary Fig. S6). It is not surprising that ActiveDriver performed relatively better in the PMH benchmark than in CGC and NCG benchmarks since some genes in the PMH list were from the predictions of ActiveDriver that potentially favors the performance of ActiveDriver. In addition to better overall performance, our methods also performed consistently better than other tools in most individual tumor types (Supplementary Figs S4–S6).

Although it downplays the importance of precision and overemphasizes the importance of recall, *F*1-measure has been used by some previous tools (Guo *et al.*, 2019). Consistently, the relative performance of these prediction tools remained largely unchanged using the *F*1-measure compared to the F0.2-measure; WMDS.netP and WMDS.net are still the best performers among these tools (Supplementary Fig. S7). Taken together, these comparisons suggest our method is powerful among various datasets and outperforms the other top-tier tools.

### 3.4 Mutation frequency of driver genes predicted by WMDS.net

**Fig. 4.**
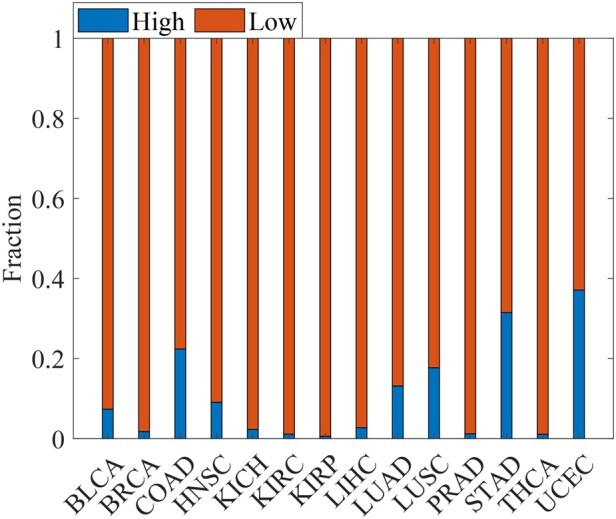
Fraction of driver genes with low- and high-mutation frequency for 14 types of cancer predicted by WMDS.net in the CCG gene list. Low mutation: somatic mutation frequency <0.05 in tumor samples; high mutation: somatic mutation frequency >0.05 in tumor samples

‘Long-tail phenomenon’ states that cancer driver genes consist of a small number of frequently mutated genes and a large number of infrequently mutated genes (Reimand and Bader, 2013). Discovering driver genes with rare mutation frequency in the long tail of genetic alterations remains challenging. Although WMDS.net identifies cancer driver genes based on transcriptome networks, we can use their somatic mutation data to assess how often these identified driver genes are mutated in tumor samples. To evaluate the ability of WMDS.net to find driver genes of rare mutation, we first obtained somatic SNVs data of 14 cancer datasets from TCGA. Then, the driver genes predicted by WMDS.net that overlap with the CCG gene list were divided into driver genes with low mutation frequency (frequency <0.05) and driver genes with high-mutation frequency (frequency >0.05) according their SNVs data. We calculated the fraction of these two categories of driver genes among 14 types of cancer. As shown in Figure 4, most driver genes predicted by WMDS.net were rarely mutated among different cancer types, indicating the strong ability of WMDS.net of finding driver genes with low-frequency mutations. Here, we took TCGA LUAD cancer as an example and found that many driver genes with low mutation frequency overlapped with the CCG gene list, but not found by other methods (Supplementary Table S2). For example, ‘ERBB3’ was detected as a driver gene by WMDS.net in LUAD, but was not found by other methods. ERBB3 is rarely mutated in LUAD based on the cosmic database (https://cancer.sanger.ac.uk). ERBB3 encodes a member of the epidermal growth factor receptor family of receptor tyrosine kinases and plays an important role in several cancers (Gandullo-Sanchez *et al.*, 2022).

### 3.5 Personalized driver genes predicted by WMDS.net

**Fig. 5.**
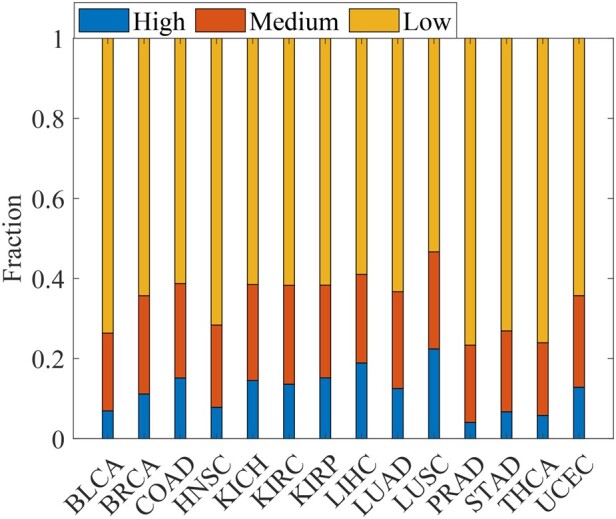
Fraction of personal driver genes in 14 cancer data sets. High-frequency personalized drivers are genes predicted as drivers in more than 60% of patients in a cancer type (i.e. with frequency ≥0.6), the medium-frequency personalized drivers with 0.3≤frequency <0.6 and low-frequency personalized drivers with frequency <0.3

We also applied WMDS to the personalized patient-specific networks constructed by SSN (Liu *et al.*, 2016) (Supplementary Fig. S2) to find personalized driver genes for each cancer patient. For a given type of cancer, we defined the high-frequency personalized driver gene that was predicted as a driver in more than 60% of patients in this cancer type (i.e. frequency ≥0.6), the medium-frequency personalized driver gene (0.3≤frequency <0.6) and low-frequency personalized driver gene (frequency <0.3). As shown in Figure 5, the low-frequency personalized driver genes account for a large proportion of driver genes among all types of cancer. That is, each patient carries its own unique set of cancer driver genes, indicating the unexpectedly high tumor heterogeneity of cancer patients in the same type of cancer. Furthermore, these personalized driver genes were often not detected by methods based on cancer-type-wide gene co-expression networks. For instance, ‘CDKN2A’ was identified as a personalized driver gene in LUAD by WMDS.netP, but not identified by WMDS.net (Supplementary Table S3). CDKN2A is known to be an important tumor suppressor gene in several cancer including LUAD (Nahar *et al.*, 2018).

### 3.6 Running time of WMDS

The time complexity of computing WMDS is the same as that of MDS. We implemented the WMDS algorithm using Matlab in a workstation with Intel 4 CPU (3.40 GH×4) and 24 GB RAM. For a network with about 10 000 nodes, the algorithm can produce results within 1 min.

## 4 Discussion

Network controllability is a basic concept of control theory, which quantifies the ability of steering a system from any initial state to any final desired state in finite time (Hu *et al.*, 2019; Liu *et al.*, 2011; Muller and Schuppert, 2011). The perturbance of state on one node can influence and change the state of the adjacent nodes through their interactions. A set of nodes that are known as driver nodes can effectively control complex networks through their external input and their intrinsic interactions with neighboring nodes in the network (Lombardi and Hornquist, 2007). Therefore, it is of great theoretical and practical significance to study the controllability of complex biological network systems from the perspective of network theory.

For the transcriptional co-expression network, its network controllability can be interpreted by operating a few key regulators to guide the transcriptional program from one state to another. Finding the key regulators in the transcriptional program can provide key insights into the network state transition underlying cellular phenotypes. To address this challenge, here, we proposed to identify the key regulators in the transcriptional network as a MDS of driver nodes that can fully control the network state transition (Nacher and Akutsu, 2012). Based on the theory of structural controllability, we applied a WMDS network model to find the driver nodes of differential co-expression networks (denoted as WMDS.net). The WMDS.net algorithm integrates the degree of nodes in the network and the significance of gene co-expression difference between two physiological states.

Cancer is a genetic disease that is driven by aberrant genetic and epigenetic changes (Meyerson *et al.*, 2010; Stratton *et al.*, 2009). During the progression from a normal state to a tumor state, cells often undergo drastic rewiring of the transcription co-expression networks. We applied the WMDS.net to predict key nodes as cancer driver genes that control the state transition of gene co-expression networks in the process of tumorigenesis and cancer progression. A total of 6835 specimens among 14 major types of cancer in TCGA, each of which contained at least 15 normal samples, were used for detecting driver genes. Our comprehensive evaluation demonstrated that genes predicted by WMDS.net are significantly more enriched than other top-tier tools in the CGC, NCG and PMH databases that provide approximate benchmarks for real cancer-associated genes.

From the perspective of detecting tumor driver genes, WMDS.net provides several advanced features compared with currently existing methods. First, WMDS.net gives a better balance between precision and recall of predicted driver genes. The number of predictions by WMDS.net is moderate, unlike some tools (e.g. iPAC and PNC) that are ambitious to predict too many drivers (e.g. >3000) (Guo *et al.*, 2019; Ryslik *et al.*, 2013) or other tools (e.g. DriverNet) that are too conservative to predict too few drivers (e.g. <10) (Bashashati *et al.*, 2012). It is also worth noting that although both WMDS.net and PNC are network-based methods, the determination of the minimum driving nodes by the PNC algorithm in an undirected network is equivalent to selecting at least one endpoint from each edge with two endpoints in the graph, so that it will result in too many nodes being identified as driver genes (Supplementary Material). Second, unlike some tools that only focus on genes with high-mutation rate and thus miss some real driver genes (Lawrence *et al.*, 2013; Tamborero *et al.*, 2013b), WMDS.net has a strong ability of identifying driver genes with low mutation rate. Third, WMDS.net is also applicable to individualized differential co-expression networks to find personalized cancer drivers for individual cancer patients. Notably, the personalized cancer driver genes of different patients are quite different, indicating the high tumor heterogeneity of cancer patients in the same type of cancer. The discovery of cancer driver genes at both the population and individual levels will not only fundamentally improve the understanding of carcinogenesis, tumor promotion and progression, but also inform potential therapies targeted against dysregulation of driver genes.

Several caveats for our study should be acknowledged. First, WMDS.net is based on the collected known molecular interaction networks. Such networks are usually incomplete with some unknown or questionable interactions, and they are also not tissue-specific, which may lead to inaccurate prediction of the key regulators that are really involved in context-dependent transcriptional regulation. However, we verified that our method of constructing differential co-expression network is robust and stable for different sample sizes (Supplementary Fig. S1). Taking BRCA as an example, 35, 40 and 45 tumor samples were used to construct differential co-expression networks. Compared with the 40-sample network, the edges recurrence rates of 35- and 45-sample networks were 94.67% and 96.20%, respectively. Second, WMDS.net is based on undirected co-expression networks and only utilizes the expression alteration information of genes and the relationship between them; however, this may not be true for all biological networks. The WMDS model provides approximate equivalence to the structural controllability theory when the edges of the MDS are bi-directional. In the future, it is worth studying how to integrate directed biological information, such as transcription factor regulation (e.g. non-linear dynamics and negative feedback loop) and network topology information to discover a complete catalog of key regulators that can steer a complex biological system from normal state to pathological state or *vice versa*. Finally, our method tended to select the MDS with members having the highest degree and most significant difference of co-expression correlation in a given network. There are also many examples in which driver nodes are not hubs in a network (Liu *et al.*, 2011). Although our method facilitates the detection of driver nodes with high connectivity, it may miss the detection of non-hub driver nodes in gene co-expression networks.

In summary, WMDS.net is an innovative network control tool for identifying driver nodes that can guide the state transition of transcriptional co-expression network. It helps to reveal the key regulators of cellular processes in both physiological and pathological states. To confirm its validity, we applied WMDS.net to the discovery of cancer driver genes in TCGA RNA-seq datasets. WMDS.net is powerful among various cancer datasets and outperformed the other top-tier tools with a better balance between precision and recall.

## Supplementary Material

btad071_Supplementary_Data

## Data Availability

The source codes for implementing WMDS.net are deposited at https://github.com/chaofen123/WMDS.net.
