# Supplementary material for: WMDS.net: a network control framework for identifying key players in transcriptome programs
Source: Bioinformatics. 2023 Feb 2;39(2):btad071. doi: 10.1093/bioinformatics/btad071 (PMC9925106; doi:10.1093/bioinformatics/btad071)
Supplement: btad071_Supplementary_Data [file btad071_supplementary_data.pdf]

WMDS.net: a network control framework for identifying key players in transcriptome programs

Xiang Cheng, Amanullah Md, Weigang Liu, Yi Liu, Xiaoqing Pan, Honghe Zhang, Haiming Xu, Pengyuan Liu, Yan Lu

Supplementary Data

Supplementary Materials

PNC algorithm is based on Feedback Vertex Set (FVS)-based control (FC) method instead of the MDS proposed by Nacher. PNC developers (Guo, et al., 2019) proposed a graph-theoretic algorithm called Nonlinear Control of Undirected network Algorithm (NCUA) based on the nonlinear FC framework for determining the minimum driver nodes in undirected networks.

Firstly, directed FVS (DFVS) is a classical method to study network control in directed networks (Zanudo, et al., 2017). DFVS find FVS in a directed network. A subset of the vertex set of a graph is a FVS of the graph if the resulting graph is acyclic after removing the vertex subset from the graph. DFVS is not appropriate for analyzing gene networks as they are undirected. Furthermore, the authors of PNC assumed that each edge in an undirected network is bi-directional and that each bi-directional edge is considered as a feedback loop, then they simply applied DFVS to the undirected network, which they referred to as NCUA. NCUA searches the sets of minimum nodes whose removal leaves the graph without edges in undirected networks. Therefore, from the intuition of the NCUA definition, we can see that NCUA is simply equivalent to selecting at least one endpoint from each edge with two endpoints.

Secondly, the network controllability theory described in the PNC paper is as follow: "we select the driver nodes by solving the following ILP:

$$minf = \sum_{v \in V_T} y_v$$

$$s.t. \sum_{\{v,u\} \in E_1} y_v \geq 1 \ (every\, u \ \in \ V_\perp), y_v \ \in \{0,1\}$$

where $\sum_{v \in V} y_v$ denotes the number of candidate nodes and y_v is an indicative variable; $\{v, u\} \in E_1$denotes the edge connecting V_v and V_u in the bipartite graph G (V_T, $V_\perp$, E_1); when node v in the up side node set in the bipartite network is within the driver nodes, y_v= 1, and otherwise, y_v= 0."

Similarly, according to ILP we can also conclude that their method of selecting driver nodes is equivalent to selecting at least one endpoint from each edge with two endpoints. Therefore, it seems inefficient to simply apply this DFVS method to this undirected network problem. Using the DFVS-based method, PNC resulted in identifying too many nodes (as driver genes) in order to fully control the whole network. Indeed, it's true from the results that PNC selected as many as 3000 genes as driver genes in gene co-expression networks.

References:

Guo, W.F., *et al.* A novel network control model for identifying personalized driver genes in cancer. *Plos Computational Biology* 2019;15(11).

Zanudo, J.G.T., Yang, G. and Albert, R. Structure-based control of complex networks with nonlinear dynamics. *Proc Natl Acad Sci U S A* 2017;114(28):7234-7239.

Table S1. Summary of TCGA cancer data sets used in this study.

Abbreviations	Full name	No. of normals	No. of tumors
BLCA	Bladder urothelial carcinoma	19	301
BRCA	Breast invasive carcinoma	113	1064
COAD	Colon adenocarcinoma	50	453
HNSC	Head and neck squamous cell carcinoma	44	482
KICH	Kidney chromophobe	24	66
KIRC	Kidney renal clear cell carcinoma	72	526
KIRP	Kidney renal papillary cell carcinoma	32	222
LIHC	Liver hepatocellular carcinoma	50	297
LUAD	Lung adenocarcinoma	59	488
LUSC	Lung squamous cell carcinoma	49	428
PRAD	Prostate adenocarcinoma	52	379
STAD	Stomach adenocarcinoma	32	415
THCA	Thyroid carcinoma	58	508
UCEC	Uterine corpus endometrial carcinoma	35	517

Table S2 Driver genes with low-mutation frequency detected by WMDS.net but not detected by other methods in LUAD

ABL1	DEK	HOXD11	NTRK1	STAT6
ABL2	DNAJB1	HOXD13	NUP98	SUFU
ACVR2A	DNM2	HRAS	PAX8	SYK
AKT1	DNMT3A	HSP90AA1	PDGFRB	TCF3
AR	EBF1	IL6ST	PIK3R1	TCF7L2
ARHGEF12	EIF4A2	JAK1	PLCG1	TERT
ARNT	ELL	JUN	PML	TGFBR2
ATF1	ELN	LCK	PMS2	TNFRSF14
ATR	EP300	LCP1	POT1	TOP1
BCL2	EPAS1	LEF1	PPARG	TSC1
BIRC3	EPS15	LMNA	PPP2R1A	TSHR
BLM	ERBB3	LMO1	PRKACA	VHL
BMPR1A	ESR1	LMO2	PTPN11	VTI1A
BRCA1	ETV1	LYL1	RAC1	WWTR1
CASP8	ETV4	MAP2K4	RAD21	XPO1
CBL	EWSR1	MAP3K1	RAF1	YWHAE
CD74	EXT1	MAPK1	RALGDS	ZBTB16
CD79A	FAS	MITF	RARA	
CDH1	FBXW7	MLH1	REL	
CDK4	FGFR4	MYB	RHOA	
CDK6	FOXA1	MYC	RHOH	
CEBPA	FOXO1	MYD88	SDC4	
CHD4	FOXO4	MYOD1	SF3B1	
CIC	FUBP1	NACA	SFPQ	
CLTC	FUS	NCOA1	SMAD2	
CREB1	GNA11	NCOA2	SMAD3	
CREBBP	GNAQ	NFKB2	SMARCB1	
DCTN1	HIF1A	NOTCH1	SMARCE1	
DDIT3	HNF1A	NPM1	SRC	
DDX5	HOXA9	NSD1	STAT3	

Table S3 Personalized driver genes detected by WMDS.netp but not detected by other methods in LUAD

BRCA2
CARD11
CBLC
CDKN2A
DAXX
ERG
EZH2
EZR
FANCA
FGFR3
HMGA1
IDH2
MAP2K1
MDM2
NF2
RECQL4
TAL1
U2AF1

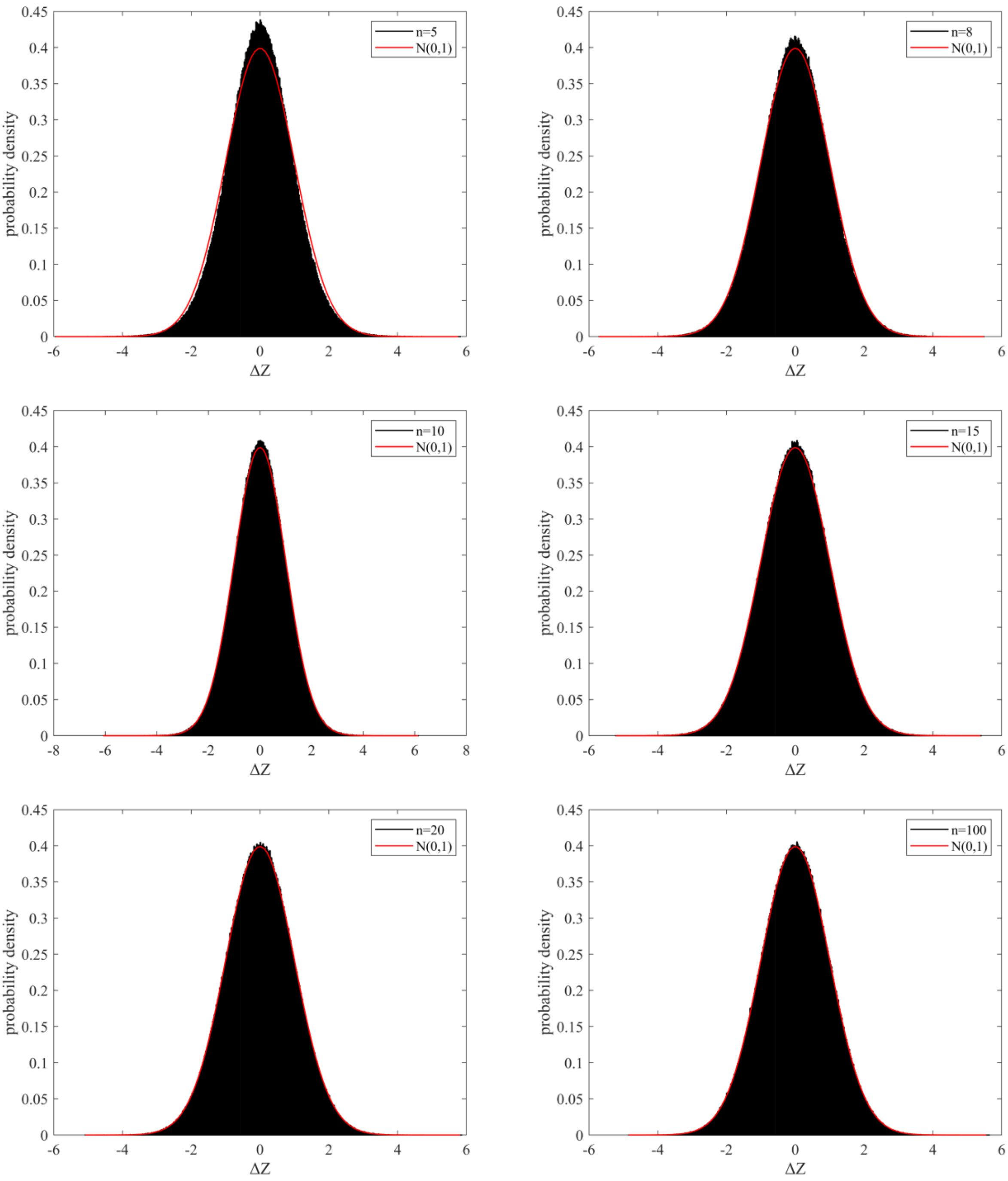

Figure S1. The distribution of ΔZ under different sample sizes. The distribution of ΔZ under the null hypothesis was simulated by randomly generating two pairs of sequences with sample size (n) of 5, 8, 10, 15 and 20, 100 for 2,000,000 times. The distribution of ΔZ is a good approximation of standard normal distribution when n > 15. Kolmogorov-Smirnov test was performed to examine the difference between the simulated distribution and the standard normal distribution using the Matlab function 'lillietest' with 10000 times of random sampling. P-values from the Kolmogorov-Smirnov test were 0.0054, 0.0194, 0.0289, 0.1056, 0.2849, and 0.5000 for sample sizes of 5, 8, 10, 15, 20 and 100, respectively. When the p-value > 0.05, we cannot reject the null hypothesis that the data come from a normal distribution. Therefore, we chose TCGA cancer types containing more than 15 normal samples to apply our method.

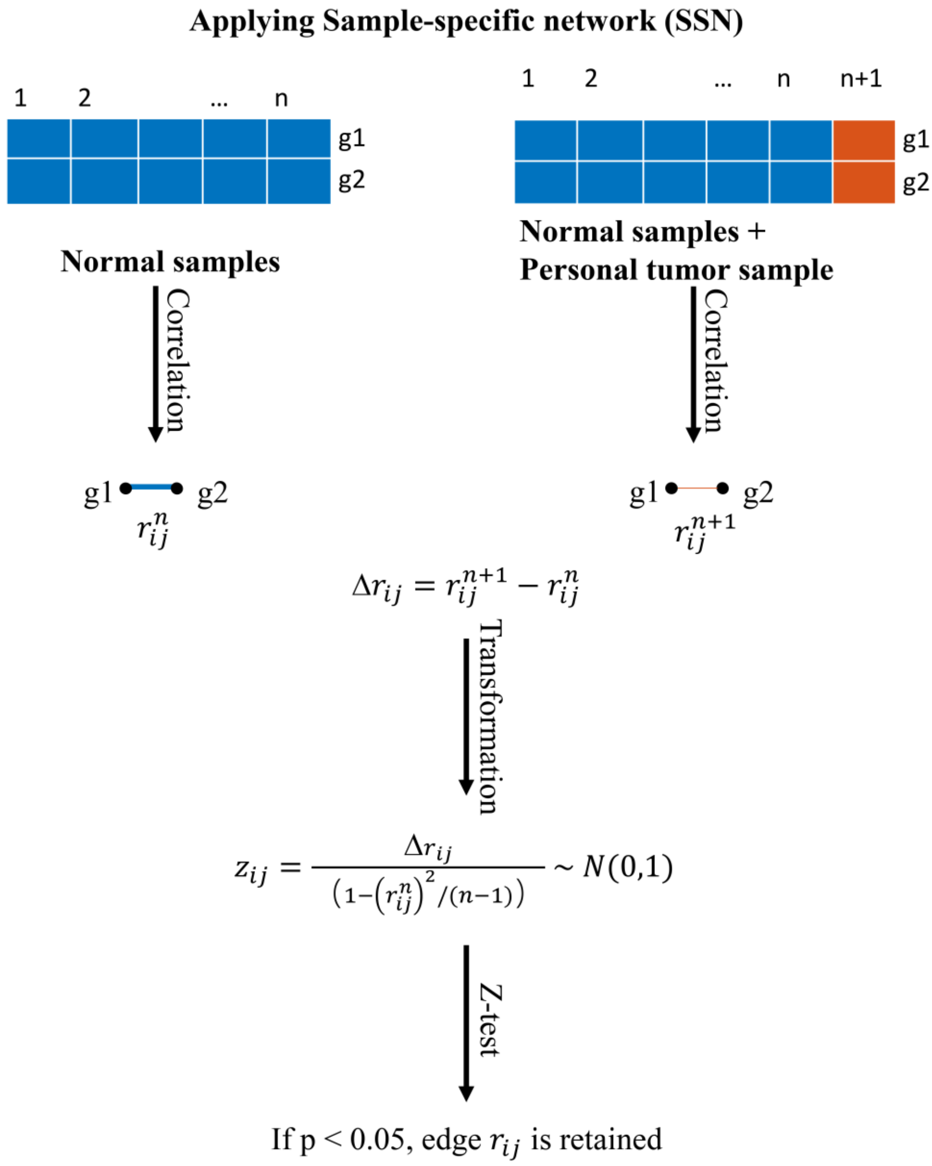

Figure S2. Construction of sample specific network (SSN). The SSN method (Liu, et al., 2016) was applied to the construction of "personalized" network. Briefly, we first identified a group of paracancerous normal tissue samples for the studied cancer type. The correlation of each pair of genes was calculated according to the expression data of these samples to construct the co-expression reference network. Then, an individual tumor sample from a cancer patient was added to the reference samples, and the correlation of each pair of genes was re-calculated to construct a co-expressive perturbed network with this added sample. The difference of the corresponding edge between co-expression reference network and co-expression perturbed network, i.e., Δr_{ij}, was calculated. The significance of Δr_{ij} was examined by Z-test. Finally, all the edges with significantly differential correlations (i.e., p-value < 0.05 for Δr_{ij}) were retained and used to construct the personalized differential co-expression network for that cancer patient.

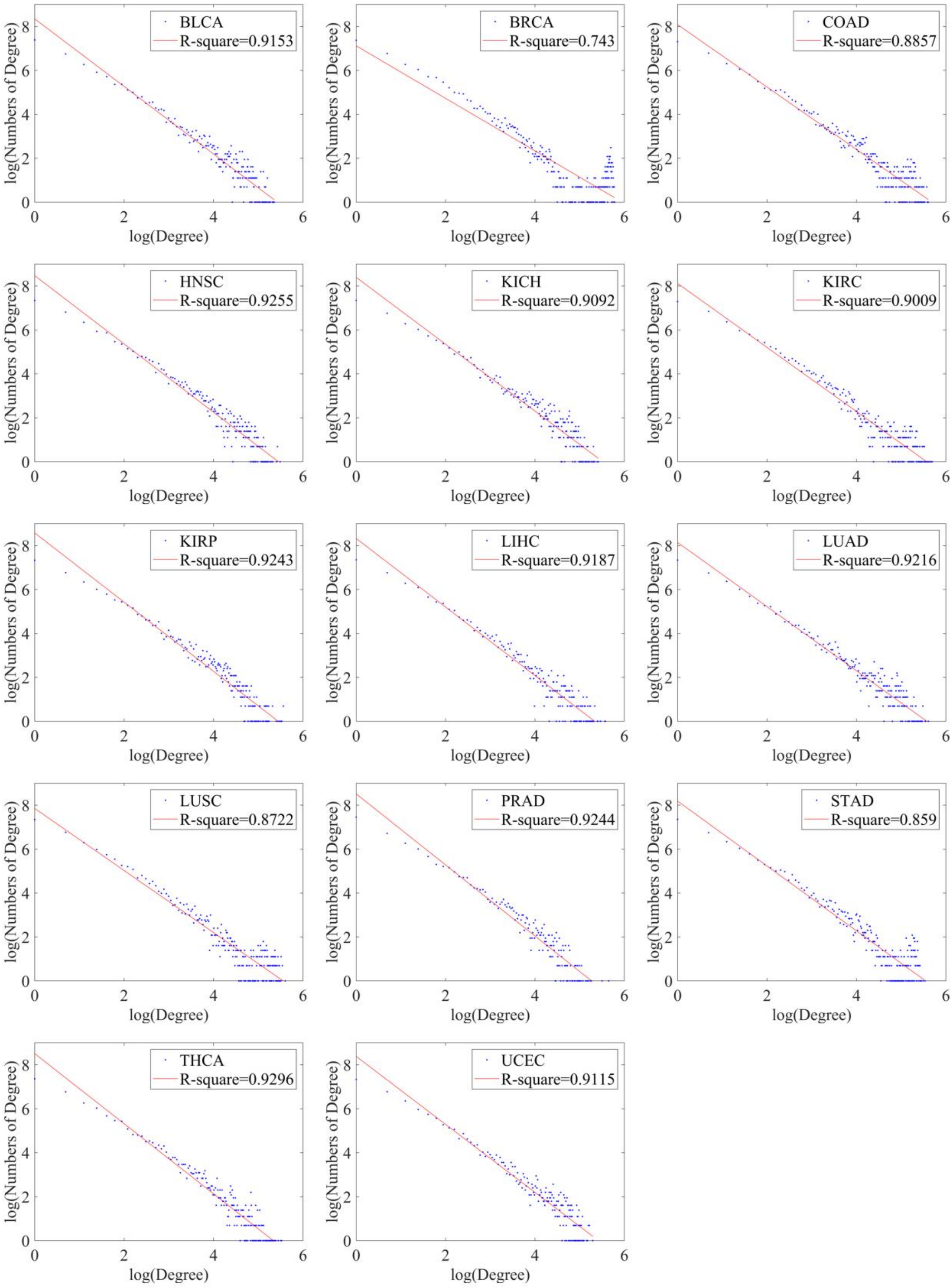

Figure S3. Degree distribution of network nodes in each of 14 cancer types from TCGA. In each cancer type, log2(numbers of degree) versus log2(degree) for the constructed differential co-expression networks was plotted and its associated fitting R-square of the linear regression model was calculated.

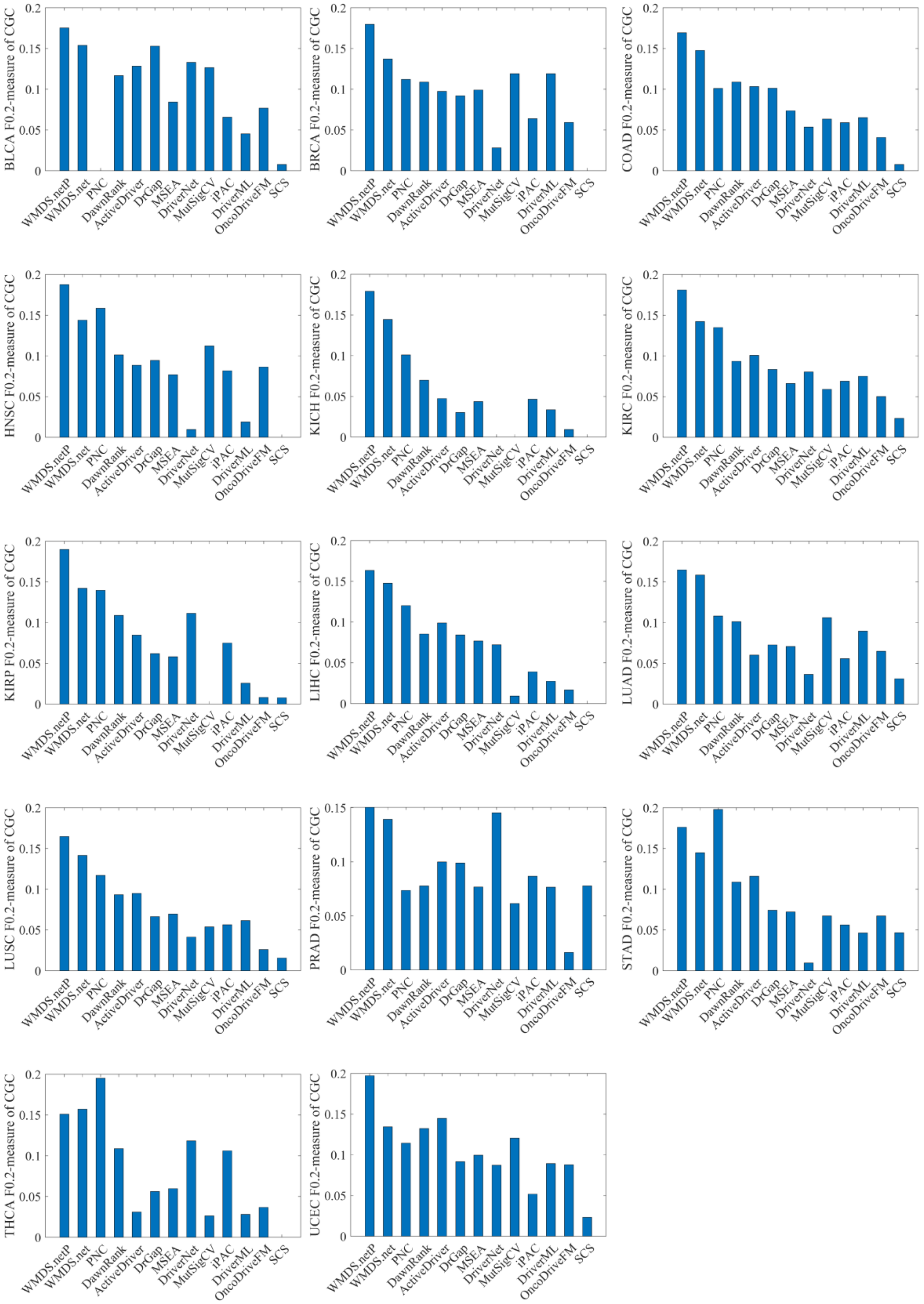

Figure S4. F0.2-measure for each method in 14 cancer types according to the CGC benchmark.

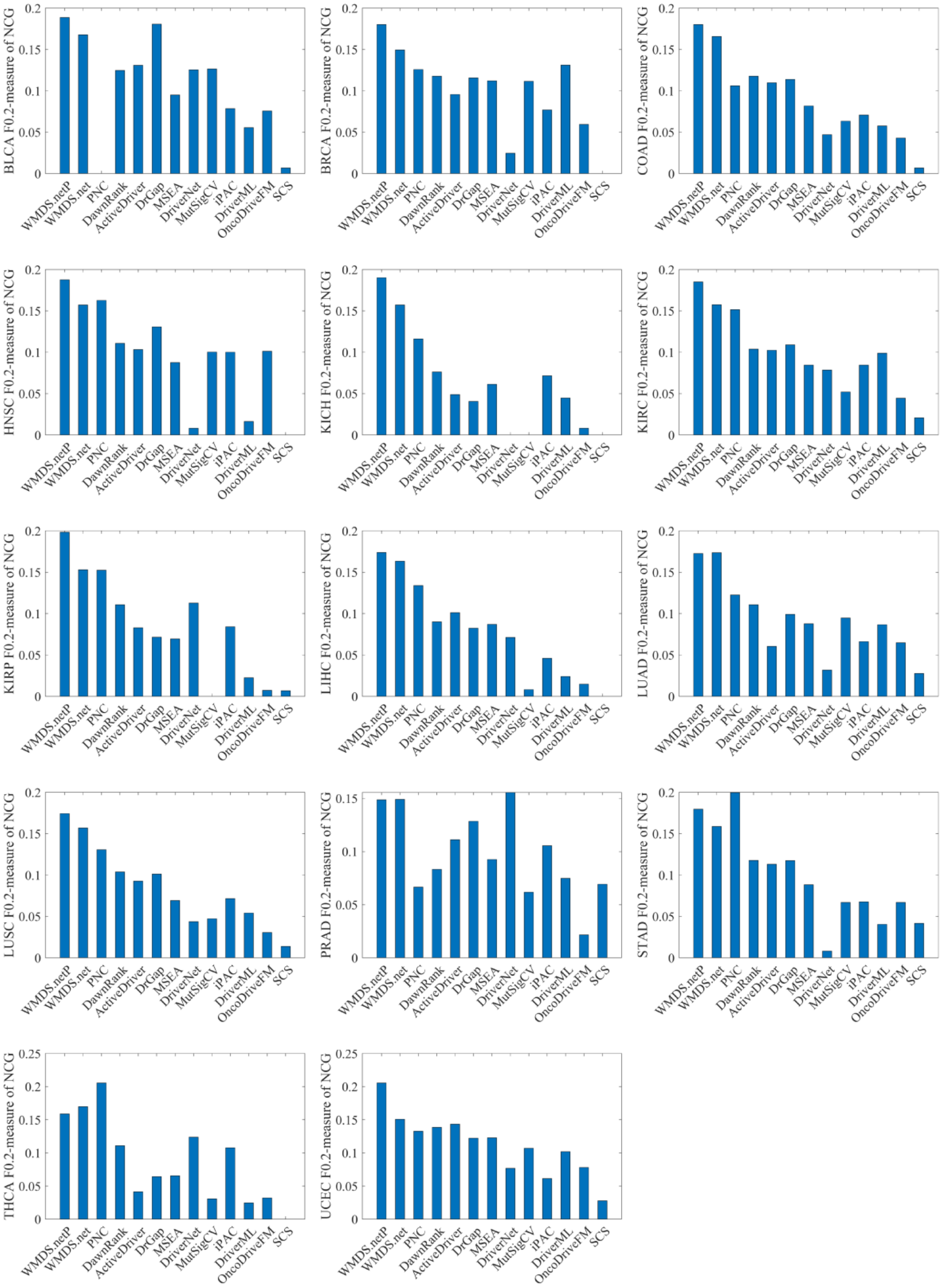

Figure S5. F0.2-measure for each method in 14 cancer types according to the NCC benchmark.

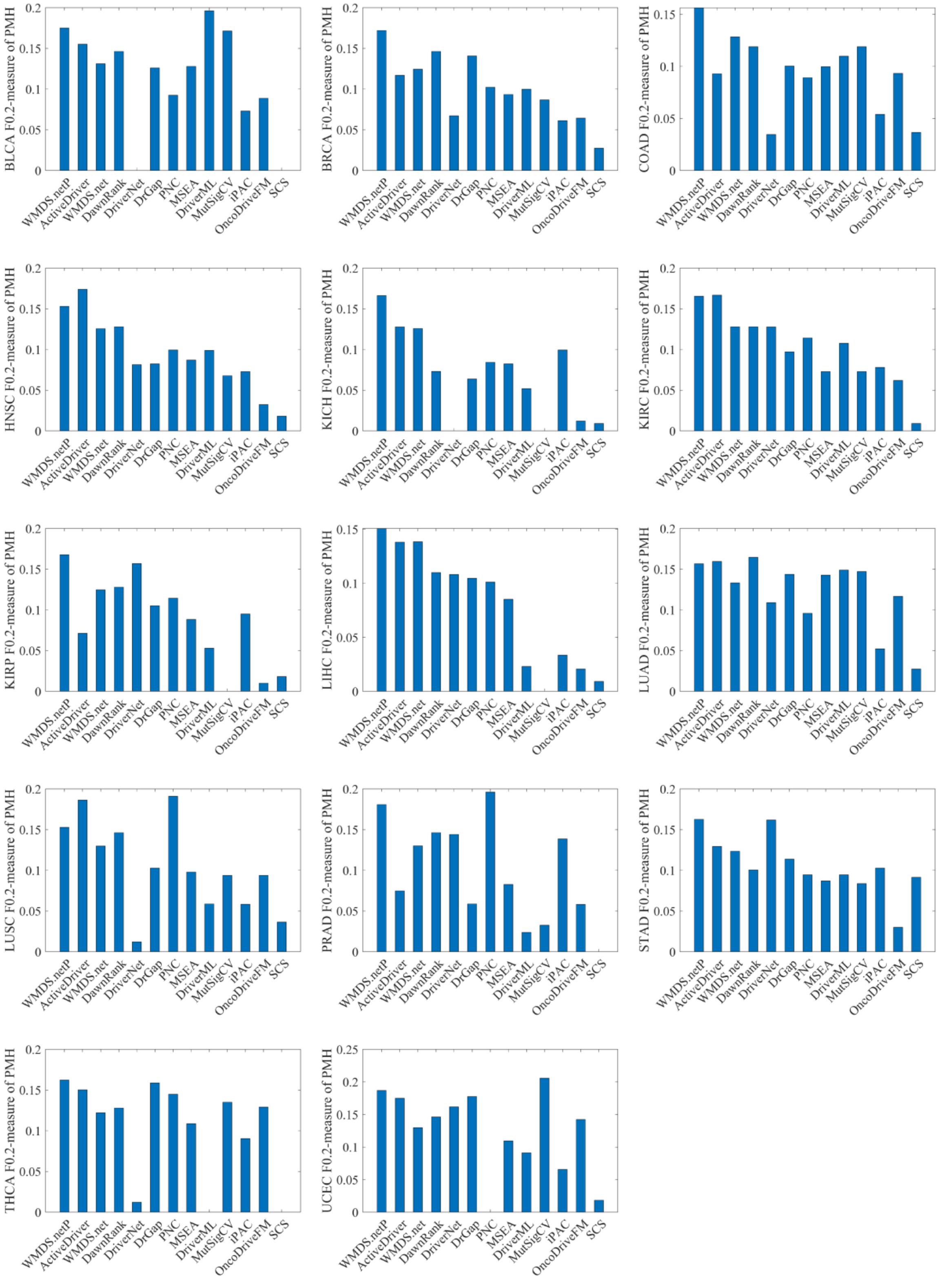

Figure S6. F0.2-measure for each method in 14 cancer types according to the PNC benchmark.

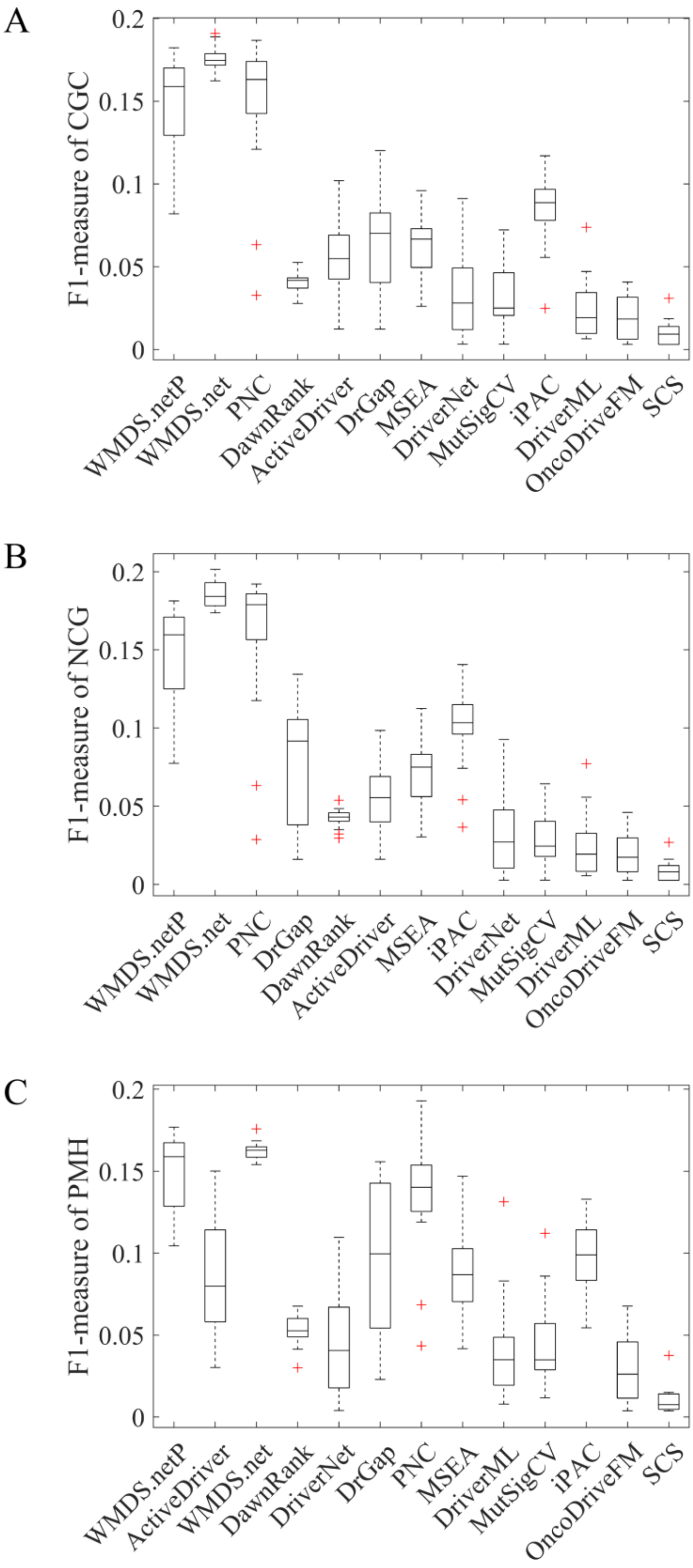

Figure S7. F1-measure of predicted driver genes in 14 TCGA datasets. (**A**) CGC benchmark. (**B**) NCG benchmark. (**C**) PMH benchmark. Tools were ordered by their median F1-measure of predicted drivers in each benchmark among 14 cancer types.
